# Morphology Effects on Electro- and Photo-Catalytic Properties of Zinc Oxide Nanostructures

**DOI:** 10.3390/nano13182527

**Published:** 2023-09-09

**Authors:** Yevgeniya Y. Kedruk, Alessandra Contestabile, Juqin Zeng, Marco Fontana, Marco Laurenti, Lesya V. Gritsenko, Giancarlo Cicero, Candido F. Pirri, Khabibulla A. Abdullin

**Affiliations:** 1 Department of General Physics, Satbayev University, Almaty 050013, Kazakhstan; y.kedruk@satbayev.university; 2 Department of Applied Science and Technology, Politecnico di Torino, 10129 Turin, Italy; alessandra.contestabile@polito.it (A.C.); marco.fontana@polito.it (M.F.); marco.laurenti1986@libero.it (M.L.); giancarlo.cicero@polito.it (G.C.); fabrizio.pirri@polito.it (C.F.P.); 3 Center for Sustainable Future Technologies @Polito, Istituto Italiano di Tecnologia, 10144 Turin, Italy; 4 National Nanotechnology Laboratory of Open Type, Al-Farabi Kazakh National University, Almaty 050040, Kazakhstan; kh.abdullin@physics.kz

**Keywords:** chemical precipitation, calcination, microwave-assisted route, zinc oxide, electrocatalyst, photocatalyst, CO_2_ reduction reaction, rhodamine-B

## Abstract

Environmental problems are among the most pressing issues in the modern world, including the shortage of clean drinking water partially caused by contamination from various industries and the excessive emission of CO_2_ primarily from the massive use of fossil fuels. Consequently, it is crucial to develop inexpensive, effective, and environmentally friendly methods for wastewater treatment and CO_2_ reduction, turning them into useful feedstocks. This study explores a unique method that addresses both challenges by utilizing ZnO, which is recognized as one of the most active semiconductors for photocatalysis, as well as a cost-effective electrocatalyst for the CO_2_ reduction reaction (CO_2_RR). Specifically, we investigate the influence of the morphology of various ZnO nanostructures synthesized via different low-cost routes on their photocatalytic properties for degrading the rhodamine-B dye (RhB) and on their electrocatalytic performance for the CO_2_RR. Our results show that the ZnO lamella morphology achieves the best performance compared to the nanorod and nanoparticle structures. This outcome is likely attributed to the lamella’s higher aspect ratio, which plays a critical role in determining the structural, optical, and electrical properties of ZnO.

## 1. Introduction

The rapid development of industries is one of the main causes of water pollution. Dyes from the textile, food, and printing industries are among the main pollutants released into reservoirs [1]. More than 800,000 tons of dyes are produced annually, and wastes are dumped into water bodies by industrial enterprises [2]. Generally, dyes are highly toxic and non-biodegradable due to their complex nature, which poses a serious threat to human health and to the environment [3,4]. Various traditional methods are used for wastewater treatment, such as coagulation, adsorption, ultrafiltration, ozonation, etc. Most of these methods have significant disadvantages such as the formation of toxic by-products, high cost, limited recovery, or high energy consumption [5]. In recent years, photocatalysis has been actively studied as an alternative method for the decomposition of organic contaminants. The advantages of this method include low cost, no need to create special conditions, and complete mineralization [6]. The decomposition of organic dyes using a semiconductor photocatalyst is an environmentally friendly and efficient method for wastewater treatment because appropriately designed photocatalysis allows one to obtain harmless final products, such as CO_2_, H_2_O, and inorganic salts, as a result of pollutant decomposition [7,8,9]. In recent decades, oxide semiconductors have attracted considerable attention as photocatalysts for the photodegradation of organic pollutants due to their high availability, low toxicity, and good thermal and chemical stability [10,11,12,13]. Zinc oxide (ZnO) is widely used as an efficient and inexpensive semiconductor photocatalyst for the decomposition of most organic chemicals and energy applications [14,15,16,17,18]. The high efficiency is due to the fact that ZnO has a high exciton binding energy at room temperature, equal to 60 meV and high electrical conductivity (~10^2^ Ω^−1^ cm^−1^) [19]. The size of the crystallites has a major influence on the photocatalytic activity of ZnO, which is typically understood and explained in terms of the specific surface area. Nano-sized materials have a highly specific surface, which provides a large number of active centers that favor organic molecule degradation [20,21].

Another major environmental issue is the excessive emission of CO_2_ due to the worldwide dependence on fossil fuel utilization. The transformation of CO_2_ into valuable chemicals and fuels attracts particular interest in both academic and industrial sectors. However, CO_2_ stability makes it difficult to reach a sufficiently good efficiency for the CO_2_RR, which, for the moment, remains far from practical industrial applications. The selectivity of the reaction toward a specific target product is another challenge because of the large variety of chemicals resulting from CO_2_RR (e.g., CO, formic acid, methane, ethylene, ethanol, acetic acid, and n-propanol). Hence, this reaction calls for efficient, stable, and cost-effective catalysts in order to improve the activity, selectivity, and efficiency of the process. Some bulk metals have been studied and demonstrated to catalyze the CO_2_RR, but their performance is still not sufficiently high [22]. For this reason, nanostructured metallic materials, with peculiar structural features with respect to bulk metals, have gained attention and show enhanced performance for CO_2_RR [23]. In particular, during the last few years, Au, Ag, and Cu nanostructured electrocatalysts have been investigated and have shown a faradaic efficiency of over 90% for CO production [24,25,26,27]. It was discovered that the improved activity of the catalyst is related to the grain boundaries of the oxide-derived Au or Cu nanoparticles [25,28,29]. Many studies also demonstrated that high-index facets and edge sites play key roles in CO_2_RR [30,31,32]. However, noble metals like Au and Ag are not cost-effective, and less expensive alternatives are highly desirable. Among the candidates, Zn is one of the most abundant and non-toxic materials, showing promising selectivity for CO production [33]. Lourenço et al. [33] studied ZnO of a three-dimensional flower-like architecture consisting of interleaving thin plates between 11 and 16 nm of thickness, showing good selectivity for CO_2_RR to CO. Urbain et al. [34] reported a nanosized Zn flake catalyst for solar-driven CO_2_RR to syngas with a CO/H_2_ ratio of 2. In particular, high efficiency and product selectivity in electrochemically reducing CO_2_ to CO have been achieved by changing the structural characteristics of Zn electrocatalysts. Won et al. [32] studied an electrodeposited hexagonal shape of Zn pieces catalyst and found via X-ray diffraction (XRD) analysis that the observed good selectivity for CO_2_RR to CO was positively influenced by the Zn (101) facet in the bulk phase. Rosen et al. [35] studied a nanostructured Zn dendrite catalyst, which can electrochemically reduce CO_2_ to CO via greatly enhanced catalytic activity and CO faradaic efficiency with respect to the bulk Zn counterparts. Though many different Zn/ZnO structures have been reported for CO_2_RR, there are no specific studies on the comparison of the morphology of ZnO catalysts, which could greatly influence the catalytic performance of CO_2_RR.

The present study aims at developing ZnO nanostructures for both electrocatalytic CO_2_RR and photocatalytic degradation of rhodamine-B dye (RhB). Traditionally, ZnO nanostructures are synthesized via various physicochemical methods, but many of them have disadvantages such as high cost, the need for high pressure, specialized equipment, and the use of toxic and environmentally hazardous chemicals, which leads to high energy consumption and the formation of a large amount of waste that is dangerous to the environment [36]. The present innovative research employs effective, one-step, environmentally friendly, and inexpensive synthesis routes to obtain different ZnO nanostructures. Moreover, these routes make it possible to control the size and shape of the particles, which is useful to tailor and improve their chemical, physical, electro-, and photo-catalytic properties. Lamellar, nanorod, and spherical morphologies were obtained via chemical precipitation, annealing, and a green microwave-assisted route, respectively.

For both CO_2_RR and RhB degradation, lamellar ZnO shows higher performance with respect to the other two types due to a much higher aspect ratio compared to the other two, thereby providing a larger amount of reaction sites.

## 2. Experimental Part

### 2.1. Materials

Zinc acetate dihydrate (Zn(CH_3_COO)_2_·2H_2_O, 99.9%), sodium hydroxide (NaOH, 98%), ethylene glycol (EG, 99.8%), potassium bicarbonate (KHCO_3_, 99.7%), Nafion^®^ 117 solution (5 wt.%), and isopropanol were purchased from Sigma-Aldrich (St. Louis, MO, USA) and used as received.

### 2.2. Synthesis of ZnO Powders

In this study, ZnO powders featuring three different morphologies, including rods, lamellar, and round-shaped nanoparticles (NPs), were synthesized via three environmentally friendly methods.

ZnO rods were prepared using the direct thermal decomposition method [37,38]. Cheap zinc acetate dihydrate was directly calcinated in a muffle furnace in the air at high temperatures for different durations. During calcination, the zinc acetate dihydrate was placed in a ceramic crucible with a lid. The mass of the synthesized ZnO nanorod samples (ZNP 1, ZNP 2, and ZNP 3) was approximately 1/3 of the zinc acetate salt mass. The ZNP 1, ZNP 2, and ZNP 3 samples were obtained by annealing at 700 °C for 10 h, 400 °C for 10 h, and 700 °C for 6 h, respectively.

ZnO lamellae were prepared via low-temperature chemical precipitation [39] starting from aqueous solutions of zinc acetate dihydrate 0.1 M and NaOH. The NaOH concentration varied for the different samples. Before the synthesis, zinc acetate and sodium hydroxide were separately dissolved in distilled water for 30 min. Then, the NaOH solution was added dropwise into a beaker containing the zinc salt solution at room temperature, followed by stirring the entire growth solution for 15 min. The resulting ZnO was thoroughly washed with distilled water via centrifugation and then dried for 12 h at 100 °C. Finally, the dried powders were calcined for one hour in the air at 450 °C, and the ZnO samples were obtained. The NaOH concentration was 0.4 M and 0.7 M for the ZNP 4 and ZNP 5 samples, respectively.

A green microwave-assisted route was used to synthesize ZnO nanoparticles (NP) [40,41]. Typically, 914 mg of NaOH was dissolved in 17 mL of EG and 3 mL of H_2_O to form solution 1, and 1100 g of Zn(CH_3_COO)_2_·2H_2_O was dissolved in 35 mL of EG and 5 mL of H_2_O to form solution 2. Then, solution 1 was added to solution 2 drop by drop. After 10 min of vigorous agitation, the mixture was transferred to a microwave oven (Milestone STARTSynth, Milestone Inc., Shelton, CT, USA) and irradiated for 6 min at 900 W (T_max._ = 220 °C). After cooling to ambient temperature, the precipitate was separated via centrifugation and washed twice with H_2_O and once with ethanol. The powder sample was finally obtained by vacuum drying at 60 °C overnight and was denoted as ZNP 6.

### 2.3. Physical-Chemical Characterizations

The microstructure and chemical compositions of the as-prepared ZnO catalysts were studied using field emission scanning electron microscopy (FESEM, Supra40 from Carl Zeiss, Jena, Germany) coupled an Oxford Instruments X-Max 10 mm^2^ silicon drift detector for energy-dispersive X-ray spectroscopy (EDX). Transmission Electron Microscopy (TEM) was carried out on an FEI Tecnai G2 F20 S-twin microscope, operated at 200 kV. The ZnO catalyst was dispersed in ethanol and subsequently drop-casted onto lacey carbon Cu grids prior to the TEM investigation. The photoluminescence (PL) spectra were measured at room temperature under 300 nm excitation using a Cary Eclipse spectrofluorimeter (Agilent, Santa Clara, CA, USA) in the range of 300–800 nm. X-ray diffraction analysis (XRD) was performed on the as-synthesized ZnO powder samples to investigate the crystal structure and on the tested ZnO electrodes in order to investigate the structural changes after the CO_2_RR. A Panalytical X’Pert Pro diffractometer equipped with Cu Kα radiation (λ = 1.54059 Å) was used as the X-ray source. Optical absorption spectra were studied using the PerkinElmer Lambda 35 UV-Vis spectrophotometer.

### 2.4. Electrochemical Analysis

#### 2.4.1. Electrode Preparation

Electrodes were prepared using a drop-casting method, which is demonstrated to be easy and efficient [42,43]. First, 15 mg of ZnO powder was mixed with 1 mg of carbon black S50, 67.5 µL of Nafion solution (5% wt), and 400 µL of Isopropanol by sonication for 30 min. Then, the uniform slurry was drop-casted onto a carbon paper substrate (gas diffusion layer, GDL28BC, Sigracet, Bonn, Germany). The electrodes were ready for use after drying at 40 °C overnight. The loading of ZnO was approximately 3.0 mg cm^−2^.

#### 2.4.2. CO_2_ Reduction Reaction Measurements

The electrochemical measurements were performed in a batch cell with two compartments separated by a proton exchange membrane (Nafion™ Membrane N117, Ion Power, New Castle, DE, USA). Both chambers were filled with 12 mL aqueous 0.1 M KHCO_3_ electrolyte during the test of all the samples. The solution was saturated by CO_2_ purging at a flux of 30 sccm for about 30 min before starting the CO_2_RR. During the tests, the CO_2_ flow was maintained at 15 sccm at the cathode and 10 sccm at the anode. An Ag/AgCl (1 mm, leak-free LF-1) electrode was used as a reference and, together with the tested working electrode, was positioned in the catholyte compartment. A Pt sheet was used as the counter electrode and was inserted into the anolyte. A potentiostat (CHI760D) was used to apply constant potential during the measurements. The gas products were analyzed using micro gas chromatography (µGC, Fusion^®^, INFICON, Bad Ragaz, Switzerland). The µGC was equipped with two modules, one with a 10 m Rt-Molsieve 5A column and Ar as the carrier gas, and the other with an 8 m Rt-Q-Bond column and He as the carrier gas. Each module was equipped with a microthermal conductivity detector (micro-TCD).

The faradaic efficiency (FE) values of the products were calculated as follows:

(1) $${FE}_{H_{2}\;or\;CO}=\frac{nFN_{H2\;or\;CO}}{Q},$$

where *n* is the number of electrons required to obtain 1 molecule of this product (*n* = 2 for H_2_ and CO); *N* is the amount of an identified product (number of moles, mol); *Q* is the total charge passed through the system during electrolysis (coulombs, C); *F* is the Faraday constant (96,485 C mol^−1^).

### 2.5. Photocatalytic Analysis

To study the photocatalytic activity of ZnO samples, an aqueous solution of rhodamine-B was used, prepared by dissolving 0.16 mg of RhB per 1 L of distilled water. In 112.5 mL of the dye solution, 9 mg of the synthesized ZnO sample was added with thorough stirring, followed by treatment in an ultrasonic bath for half an hour. A mercury arc lamp (LIH ULQ 14W, Herrsching am Ammersee, Germany) as a source of UV-Vis irradiation was placed in a flask containing the solution of RhB and ZnO. During UV illumination, the prepared solution was mixed with a magnetic stirrer.

## 3. Results and Discussion

### 3.1. Morphological and Structural Characterizations of ZnO Nanostructures

**Figure 1:**
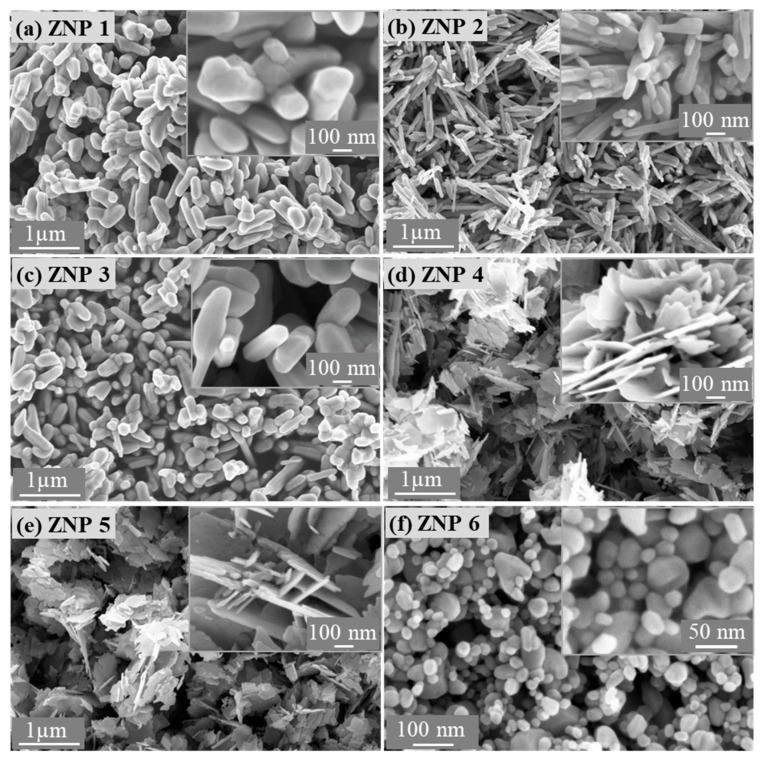
Field emission scanning electron microscope images of the ZnO samples: (**a**) ZNP 1 (annealing, 700 °C, 10 h), (**b**) ZNP 2 (annealing, 400 °C, 10 h), (**c**) ZNP 3 (annealing, 700 °C, 6 h), (**d**) ZNP 4 (precipitation, [NaOH] = 0.4 M), (**e**) ZNP 5 (precipitation, [NaOH] = 0.7 M), and (**f**) ZNP 6 (microwave-assisted synthesis).

Figure 1 shows the morphology of the ZnO powders synthesized via the three low-cost methods, whereas Table 1 provides the relevant morphological parameters measured using FESEM, as presented in Figure S1. Via calcination of zinc acetate at high temperatures, ZnO grows in the form of rods (Figure 1a–c). As the annealing time is reduced from 10 to 6 h at a synthesis temperature of 700 °C, the diameter of the rods is slightly decreased, similar to the length of the rods. When the annealing temperature is lowered from 700 °C to 400 °C with the same annealing time (10 h), the diameter of the ZnO rods is significantly reduced, whereas the length of the rods is slightly changed. In comparison, the annealing temperature has a primary influence on the growth of the rods with respect to time. Using the chemical precipitation method, it is possible to obtain ZnO in the form of lamella (Figure 1d,e). An increase in the NaOH concentration leads to a slight increase in the thickness of the flakes without significant effects on the morphology. ZnO NPs prepared with a green microwave-assisted route show a spherical form (Figure 1f) with an average diameter of approximately 40–60 nm. Table S1 summarizes the chemical compositions of all the ZnO samples obtained via EDX analysis. Zn and O are the main elements and have an atomic ratio of nearly 1. A small amount of C is associated with surface contaminations.

Table 1 shows the morphological characteristics of the ZnO samples, including the diameter, length, and ratio obtained from FESEM (Figure S1), indicating that the samples obtained via the precipitation method exhibit the highest aspect ratio. Further morphological study with TEM (Figure S2) confirms that ZNP 4 has high aspect ratio lamellar nanostructures.

**Figure 2:**
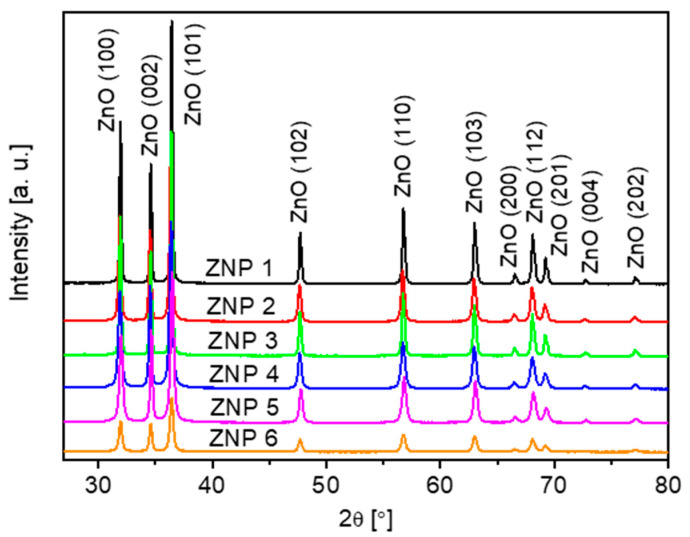
XRD patterns of the ZnO samples: ZNP 1 (annealing, 700 °C, 10 h), ZNP 2 (annealing, 400 °C, 10 h), ZNP 3 (annealing, 700 °C, 6 h), ZNP 4 (precipitation, [NaOH] = 0.4 M), ZNP 5 (precipitation, [NaOH] = 0.7 M), and ZNP 6 (microwave-assisted synthesis).

XRD measurements have been performed to better understand the crystalline structures of all the ZnO samples. As shown in Figure 2, all peaks in the XRD patterns are associated with ZnO (reference code: JCPDS 01-075-0576) without additional peaks related to impurities.

XRD analysis reveals that all the samples present a hexagonal structure (wurtzite), and the cell parameter obtained values by fitting are consistent with the theoretical values (Table 1). For sample ZNP 4, it must be stressed that the XRD results are also in accordance with the structural information obtained via electron diffraction (see Supplementary Information).

### 3.2. Photocatalytic Activity of ZnO Nanostructures for Dye Degradation

**Figure 3:**
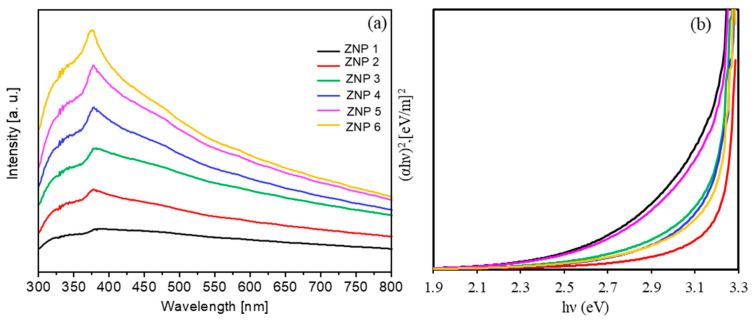
(**a**) Optical density spectra of all ZnO samples; (**b**) Tauc’s plot for energy bad gap determination.

First, we discuss the optical properties of the synthesized ZnO nanostructures. As shown in Figure 3, all ZnO nanostructures absorb light in the UV-Vis range with a maximum wavelength of 380 nm, and they are transparent in the visible region of the spectrum.

The width of the optical band gap can be estimated from the edge of the absorption band using the Tauc method [44]. The Tauc relation is shown in Equation (2),

(2) $$(\alpha h\nu )=C{\left(h\nu -E_{g}\right)}^{n},$$

where α is the absorption coefficient; C is the coefficient of proportionality; $n=\frac{1}{2}$ since ZnO is a direct-band gap semiconductor material [44], hν is the photon energy, and $E_{g}$ is the band gap width. The coefficient of proportionality C can be expressed in Equation (3),

(3) $$C=\alpha d=ln\left(\frac{I_{0}}{I}\right),$$

where d is the film thickness, *I* is the intensity of the monochromatic light that passes through the substance, and *I*_0_ is the intensity of the light incident on the absorbing layer. For the synthesized ZnO samples, the estimated optical band gap is about 3.2 eV with a relative error of 6%, which is in agreement with the literature [14,15].

The photocatalytic activity of ZnO materials for RhB decomposition was then evaluated under UV-Vis light illumination. The optical density spectra obtained during the decomposition of RhB in solutions with different ZnO samples are shown in Figure S3. The RhB solution with each ZnO material was placed under UV-Vis light illumination and sampled and analyzed every 30 min for a total duration of 150 min. The maximum absorption intensity of the initial RhB solution was about 556 nm. With an increase in the duration of the UV-Vis illumination, the absorption intensity of the RhB solution decreased, indicating that the concentration of RhB in the solution decreased accordingly. It was also noted that the absorption intensity of the RhB solution remarkably decreased during the first 30 min of exposure. Then, the decrease rate significantly dropped and reached almost zero after 150 min of illumination. This phenomenon was observed for all ZnO materials, implying that all ZnO materials can promote the degradation of RhB under UV-Vis irradiation. It is worth noting that the absorption intensity of the solutions with ZNP 4 and ZNP 5 was much lower after 60 min of exposure than that of the solutions with other ZnO materials, indicating that the former two had higher photocatalytic activity for RhB degradation. Figure S4 demonstrates the change in the color of the RhB solution with the ZNP 4 sample after 150 min under UV-Vis illumination. Due to the presence of RhB, the initial solution showed an intense fuchsia color. Under UV-Vis irradiation, the solution underwent quick color fading during the first 30 min and became almost transparent after an exposure time of 150 min. This observation is consistent with the above-mentioned optical analysis in Figure S3.

**Figure 4:**
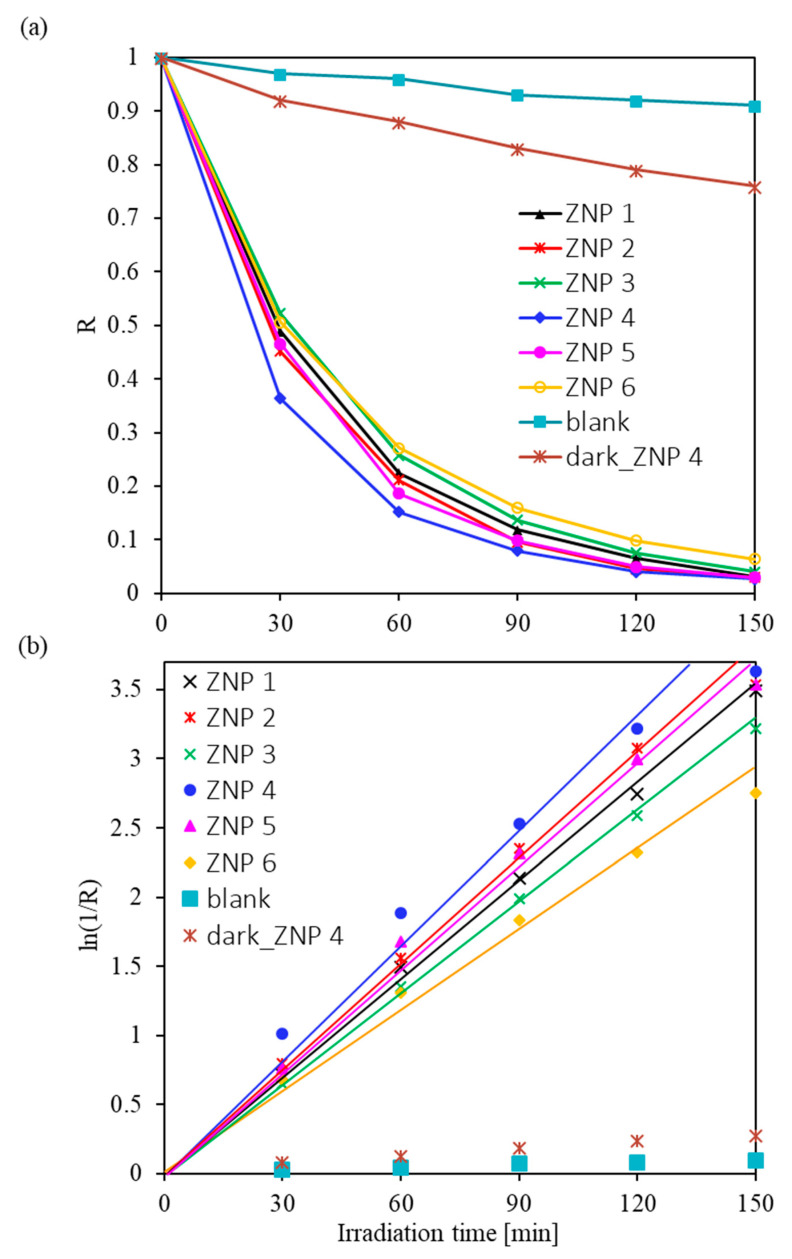
RhB degradation under UV-Vis light irradiation: (**a**) *R* (*C/C*_0_) values of RhB and (**b**) the ln(1/R) as a function of irradiation time.

The rate of photocatalytic degradation of the RhB dye in the presence of ZnO samples, k, was calculated based on the kinetic model proposed by Langmuir–Hinshelwood [45,46] in Equation (4),

(4) $$k=\frac{lnR}{-t}=\frac{ln\;(C_{0}/C)}{t},\;$$

where *C*_0_ is the initial concentration of RhB and *C* is the concentration of RhB after UV-Vis irradiation for time *t*. The percentage of dye that decomposed under UV-Vis light can be determined by Equation (5),

(5) $$R^{*}=100\left(1-R\right),$$

where *R* = *C/C*_0_. The values obtained for this coefficient and the rate of photocatalytic degradation of the dye are shown in Table S2. A high rate of RhB decomposition under UV-Vis irradiation was observed in the presence of all the synthesized ZnO samples. The rate of photocatalytic dye degradation varies from 1.23 h^−1^ (for the ZNP 6 sample) to 1.73 h^−1^ (for the ZNP 4 sample). Calculations showed that after 150 min of UV-Vis illumination of the RhB aqueous solution, 94–97% of its initial concentration decomposed. The *C/C*_0_ values of RhB and the ln(1/R) as a function of radiation time are shown in Figure 4a,b, respectively. The blank experiment showed that RhB hardly degraded after exposure to UV-Vis irradiation for 150 min, which indicates that RhB could not be decomposed without the photocatalyst. It is worth noting that ZNP 4 had the highest activity, followed by ZNP 2 and ZNP 5, while ZNP 1, ZNP 3, and ZNP 6 possessed relatively lower photoactivity. The trend of the activity of the samples corresponds well with that of the aspect ratio of the ZnO nanostructures shown in Table 1, indicating that this morphological indicator has a primary effect on the photocatalytic performance of the ZnO samples.

To explain the effect of morphology on the photocatalytic activity of the ZnO samples with respect to the RhB dye, the photoluminescence spectra of the synthesized ZnO samples were studied. As shown in Figure S5, the PL intensity of the radiative recombination through deep defect states in samples ZNP 2 and ZNP 4 reached a minimum value, indicating a reduced concentration of defects in the volume and surface of these ZnO samples. Accordingly, the lifetime of the photogenerated free carriers was longer in such samples, which could be the main reason for their higher photocatalytic activity [47,48]. It is worth noting that the ZNP 2 and ZNP 4 were in line with the best ZnO photocatalysts for similar functions, as shown in Table S3.

One of the advantages of photocatalysis is the reusability of the catalyst [49]. In this study, experiments were carried out to verify the possibility of multiple using ZnO as photocatalysts. To check ZnO reusability, the photocatalytic activity of the ZNP 4 sample was tested five times. The irradiation time for each test was 150 min. After each RhB degradation test, the solution was centrifuged to separate ZnO, which was then added to a new fresh dye solution. The initial dye concentration was the same for all the tests. Figure S6 shows that the photocatalytic activity of the ZNP 4 sample remains almost unchanged after five cycles. This result confirms that ZNP 4 photocatalyst has good recyclability properties.

The photocatalytic mechanism of the organic dye decomposition in the presence of ZnO is illustrated in Figure S7. Photocatalysis is the acceleration of a chemical reaction due to the combined action of a catalyst and light irradiation. In photogenerated catalysis, photocatalytic activity depends on the ability of the catalyst to generate electron–hole pairs, which produce free radicals that can then be involved in secondary reactions [50]. As shown in Figure S7, after light absorption, the electron–hole pairs moved toward the ZnO surface. Then, the h^+^ combined with water to produce hydroxyl radicals, whereas electrons combined with oxygen to form superoxide radical anions. Subsequently, these radicals, as oxidizing agents, reacted with the adsorbed contaminants present on the ZnO surface and decomposed them to form H_2_O, CO_2,_ and mineral acids [50,51].

### 3.3. Electrocatalytic Activity of ZnO Nanostructures for *CO_2_RR*

**Figure 5:**
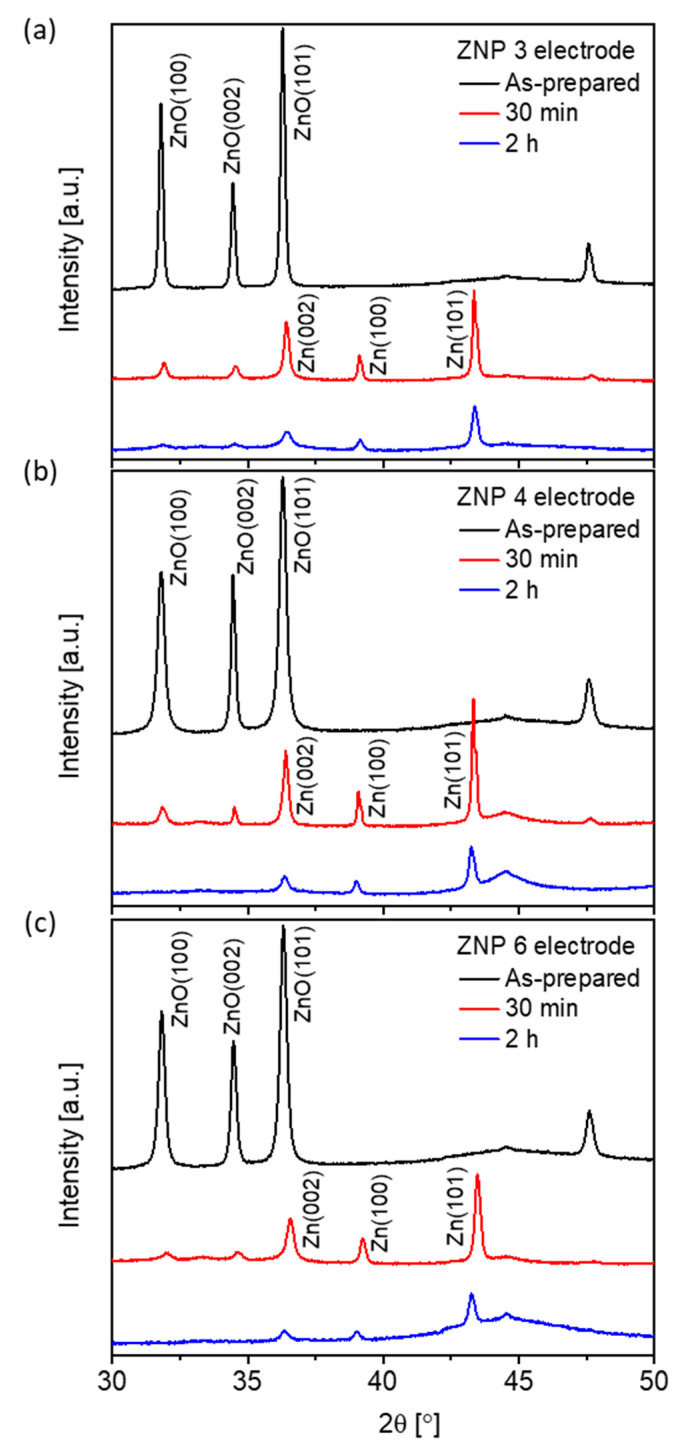
XRD patterns of the electrodes with three nanostructures: (**a**) nanorod (ZNP 3), (**b**) lamellar (ZNP 4), and (**c**) nanoparticle (ZNP 6) after different reduction time (as-prepared, after 30 min of reduction, and after 2 h of reduction at −1.0 V vs. RHE).

The electrocatalytic activity of all the ZnO samples toward the CO_2_RR was studied by CO_2_ electrolysis on ZnO electrodes in a potential-controlled mode. Under the negative potentials typically employed for CO_2_ electrolysis, ZnO is reduced to metallic Zn, as reported in the literature [41]. Figure 5 shows direct evidence that ZnO undergoes progressive reduction at −1.0 V vs. reversible hydrogen electrode (RHE), independent of the morphology of the nanostructures. The ZnO content decreased quickly in the first 30 min of reduction for all morphologies with the three representative electrodes. After 2 h of reduction, the lamellar (ZNP 4) and nanoparticle (ZNP 6) structures showed only diffraction peaks related to metallic Zn, and the nanorod (ZNP 3) structure displayed mainly metallic Zn peaks with tiny ZnO peaks, probably due to the large diameter of ZNP 3 that needed more time to be fully reduced.

**Figure 6:**
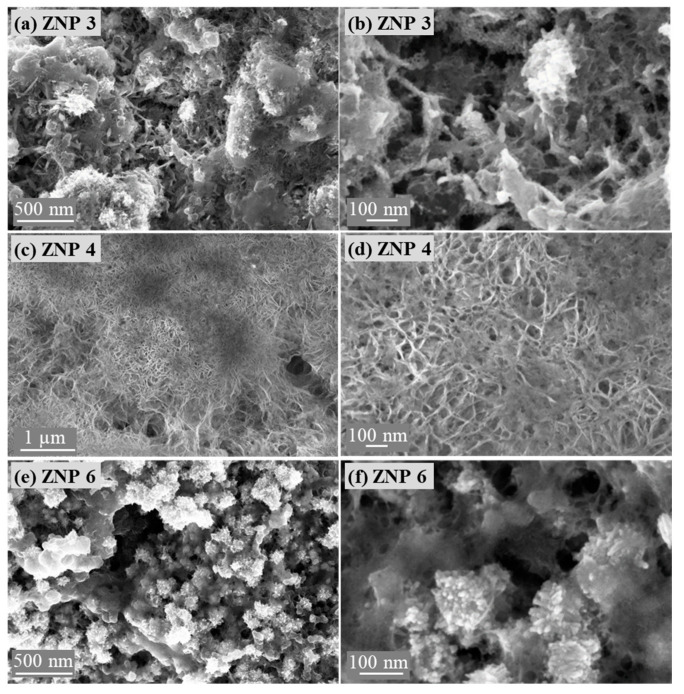
Field emission scanning electron microscope images of the reduced electrodes: (**a**,**b**) ZNP 3, (**c**,**d**) ZNP 4, and (**e**,**f**) ZNP 6.

The morphology of the reduced electrodes was also studied using FESEM. As shown in Figure 6, the nanostructures of the ZnO samples changed after being reduced to metallic Zn. All samples showed a reduced particle size. However, each electrode still retained the original morphology of the ZnO sample. ZNP 3, ZNP 4, and ZNP 6 electrodes showed nanorod-like, nanoflake, and nanoparticle morphologies, respectively. Hence, it is foreseen that the ZNP 4 electrode has a higher aspect ratio with respect to the other two electrodes.

**Figure 7:**
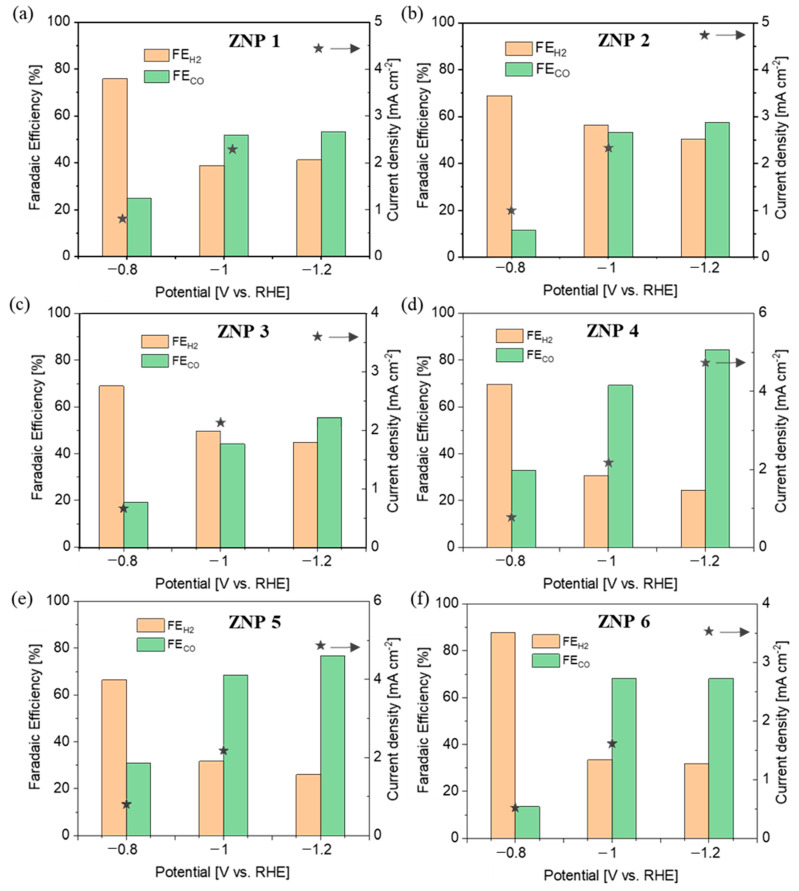
CO_2_RR performance at different potentials on various electrodes: (**a**) ZNP 1, (**b**) ZNP 2, (**c**) ZNP 31, (**d**) ZNP 4, (**e**) ZNP 5, and (**f**) ZPN 6.

In order to verify the activity and selectivity of various ZNP electrodes for the CO_2_RR, chronoamperometric measurements were carried out at various potentials of −0.8 V, −1.0 V, and −1.1 V (vs. RHE) in a three-electrode two-compartment cell using online µGC analysis. Unless specified otherwise, all the potentials refer to RHE. The gas products were analyzed during 2 h of testing at each potential, and the reported data were collected at the end of each test until the distribution of the gas products was stable. As studied via XRD analysis (Figure 5), the electrodes were mainly composed of metallic Zn derived from ZnO. The FE values of both CO and H_2_ are shown in Figure 7 for all the ZNP electrodes, together with the total current density at each applied potential.

It is evident that the electrodes featuring a lamellar structure (ZNP 4 and ZNP 5) perform far better than those with nanorod (ZNP 1, ZNP 2, and ZNP 3) and nanoparticle (ZNP 6) structures. In particular, the ZNP 4 and ZNP 5 electrodes show good CO selectivity with FE_CO_ of about 70% and 80% at −1.0 V and −1.2 V, respectively. The highest FE_CO_ is 84.3% on the ZNP 4 electrode at −1.2 V, with an appreciable current density of 4.9 mA cm^−2^. The ZNP 6 also exhibits relatively good CO selectivity and reaches FE_CO_ of about 70% at both −1.0 V and −1.2 V, outperforming the electrodes with a nanorod structure. The ZNP 1, ZNP 2 and ZNP 3 electrodes characterized by a nanorod morphology perform similarly, reaching FE_CO_ of about 50% and 60% at −1.0 V and −1.2 V, respectively. The best performance of the ZNP 4 electrode could be attributed to the higher aspect ratio of the ZNP 4 oxide material, which is preserved in the reduced electrode. It is worth noting that the ZNP 4 catalyst is in line with the best-performing ZnO catalysts reported in the literature with a batch cell setup and bicarbonate electrolyte, as shown in Table S4.

## 4. Conclusions

Various ZnO nanostructures, namely nanorods, nanoflakes, and nanoparticles, were synthesized using simple, low-cost, and environmentally friendly methods, showing an aspect ratio trend of nanoflakes > nanorods > nanoparticles.

The morphology effects on the photocatalytic activity of the ZnO nanostructures toward the degradation of RhB dye were studied in an aqueous solution under UV-Vis irradiation. It is worth highlighting that the aspect ratio of the ZnO structures was identified as a key indicator for photoactivity. By increasing the aspect ratio, the structure became more active for the photodegradation of organic dyes. Among all the investigated ZnO nanostructures, the highest rate of RhB degradation was 1.73 h^−1^ for the ZnO lamellar structure with the largest aspect ratio.

The electrochemical CO_2_RR on the ZnO electrodes was also found to be highly dependent on the morphology. The oxide-derived metallic Zn was found to be the active phase, and the difference in morphology among the various samples was conserved after ZnO reduction. It was noticed that the anisotropic lamellar morphology was the one characterized by the best performance in terms of activity and selectivity toward CO_2_RR to CO, showing a good FE_CO_ of 84.3% and a relatively high current density of 4.9 mA cm^−2^ at −1.2 V. This outcome could be attributed to the high aspect ratio of Zn derived from the lamellar ZnO structure.

In addition to the good photo- and electro-catalytical activity of the prepared ZnO nanostructures, it is also worth highlighting that all the methods employed for the synthesis of ZnO materials are economical, easy to implement, do not require complex and expensive equipment, and are appropriate for large-scale production. Hence, it is foreseen that the investigated ZnO catalysts have promising potential for implementation in industrial processes for photocatalysis and electrocatalysis.

## Figures and Tables

**Table 1:** Average dimension, aspect ratio, and cell parameters for all ZnO samples.

Sample Name	Diameter * (nm)	Length (nm)	Ratio (Length/Diameter)	Cell Parameters (Å)	
				a	c
ZNP 1	184	559	3.0	3.2502	5.2071
ZNP 2	60	550	9.2	3.2512	5.2088
ZNP 3	163	430	2.6	3.2514	5.2089
ZNP 4	23	879	38.2	3.2509	5.2079
ZNP 5	29	700	24.1	3.2513	5.2082
ZNP 6	40–60	40–60	1 (spherical shape)	3.2524	5.2109

* For the lamellar morphology, the diameter is considered as thickness.

## Data Availability

Data are available on demand.

## Supplementary Materials

The following supporting information can be downloaded at: https://www.mdpi.com/article/10.3390/nano13182527/s1, Figure S1. FESEM images of (a) ZNP 1, (b) ZNP 2, (c) ZNP 3, (d) ZNP 6, (e) (f) ZNP 4 and (g) (h) ZNP 5. Figure S2. TEM characterizaion of sample ZNP 4: bright-field TEM image (a), selected area electron diffraction (SAED) pattern (b), rotationally-averaged electron diffraction pattern (c), with peak labels corresponding to family of planes characteristic of the ZnO wurtzite crystalline structure. Figure S3. The optical density spectra of RhB solution under UV-Vis light illumination at different exposure time. The solution contained also ZnO nanostructures corresponding to samples (a) ZNP 1, (b) ZNP 2, (c) ZNP 3, (d) ZNP 4, (e) ZNP 5 and (f) ZNP 6. Figure S4. The change of color of the RhB solution with ZNP 4 for 150 min under UV-Vis illumination. Figure S5. PL spectra of the synthesized ZnO samples taken at room temperature with excitation at a wavelength of 300 nm. Figure S6. Reuse activity of ZNP 4 for RhB dye photodegradation. Figure S7. The proposed photocatalytic mechanism of ZnO nanostructures. Table S1. Element percentages obtained by EDX analysis. Table S2. Efficiency of photocatalytic degradation of dye in the presence of ZnO samples. Table S3. Comparison the performance of the herein studied ZnO with the reported ZnO photocatalysts in literature. Table S4. Comparison of Zn-based electrocatalysts for CO_2_ electrolysis in a batch cell. Refs. [21,35,41,52,53,54,55,56,57,58,59,60,61,62,63,64,65,66,67,68] are cited in Supplementary Materials.
