# Supplementary material for: Morphology Effects on Electro- and Photo-Catalytic Properties of Zinc Oxide Nanostructures
Source: Nanomaterials (Basel). 2023 Sep 9;13(18):2527. doi: 10.3390/nano13182527 (PMC10534315; doi:10.3390/nano13182527)
Supplement: Supplementary file 1 [file nanomaterials-13-02527-s001.zip › nanomaterials-2551813-supplementary.pdf]

Supporting information

Article

Morphology Effects on Electro- and Photo-Catalytic Properties of Zinc Oxide Nanostructures

Yevgeniya Y. Kedruk [1], Alessandra Contestabile [2], Juqin Zeng [2,3,*], Marco Fontana [2,3], Marco Laurenti [2], Lesya V. Gritsenko [1,4,*], Giancarlo Cicero [2], Candido F. Pirri [2,3] and Khabibulla A. Abdullin [4]

Keywords: chemical precipitation, calcination, microwave-assisted route, zinc oxide, electrocatalyst, photocatalyst, CO_2 reduction reaction, rhodamine-B.

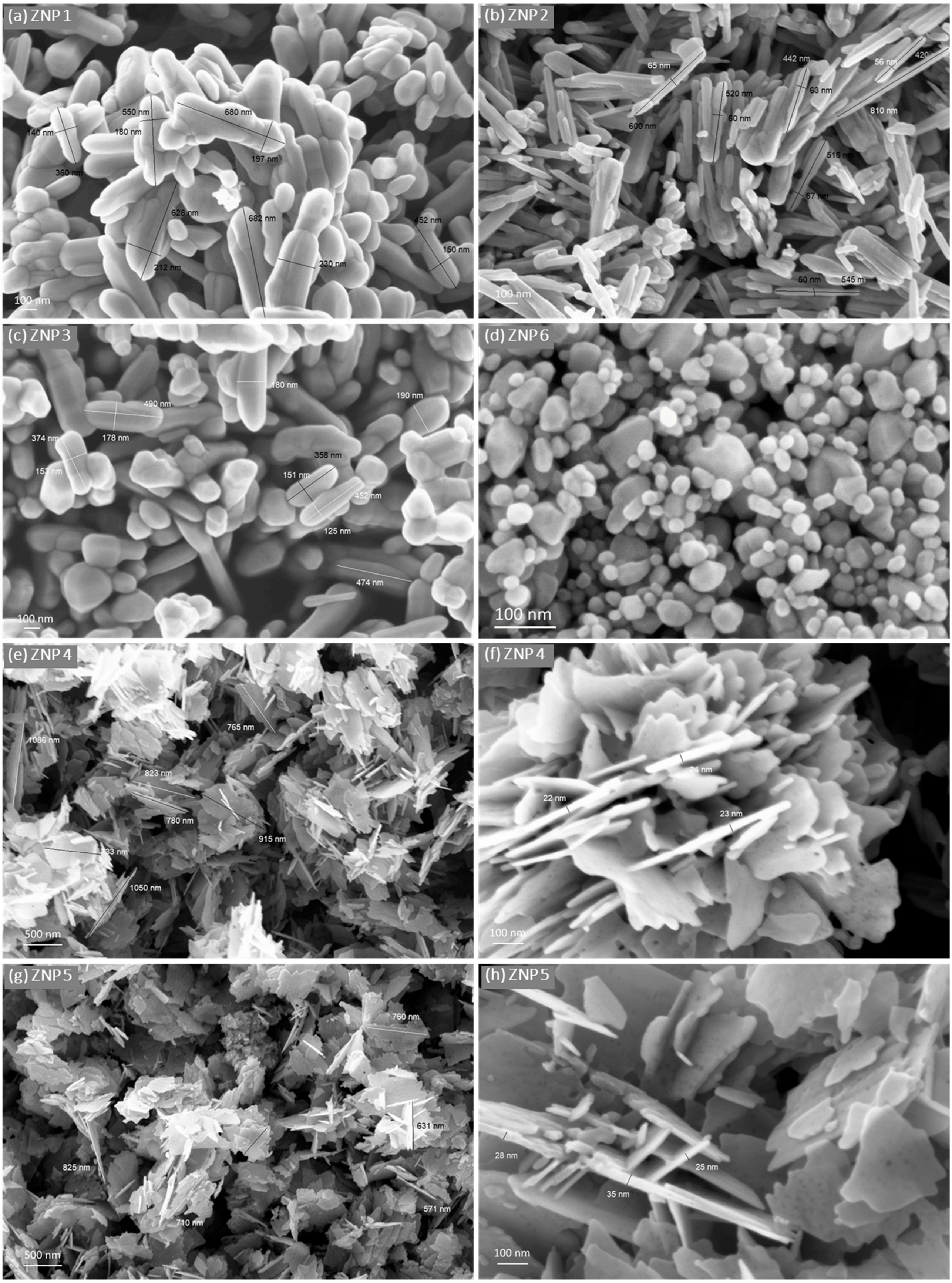

Figure S1. FESEM images of (a) ZNP 1, (b) ZNP 2, (c) ZNP 3, (d) ZNP 6, (e) (f) ZNP 4 and (g) (h) ZNP 5.

TEM CHARACTERIZATION

Figure S2 provides TEM results obtained for sample ZNP 4. By BF-TEM images such as Figure S2a, the high-aspect ratio lamellar morphology is confirmed, in accordance with the FESEM characterization.

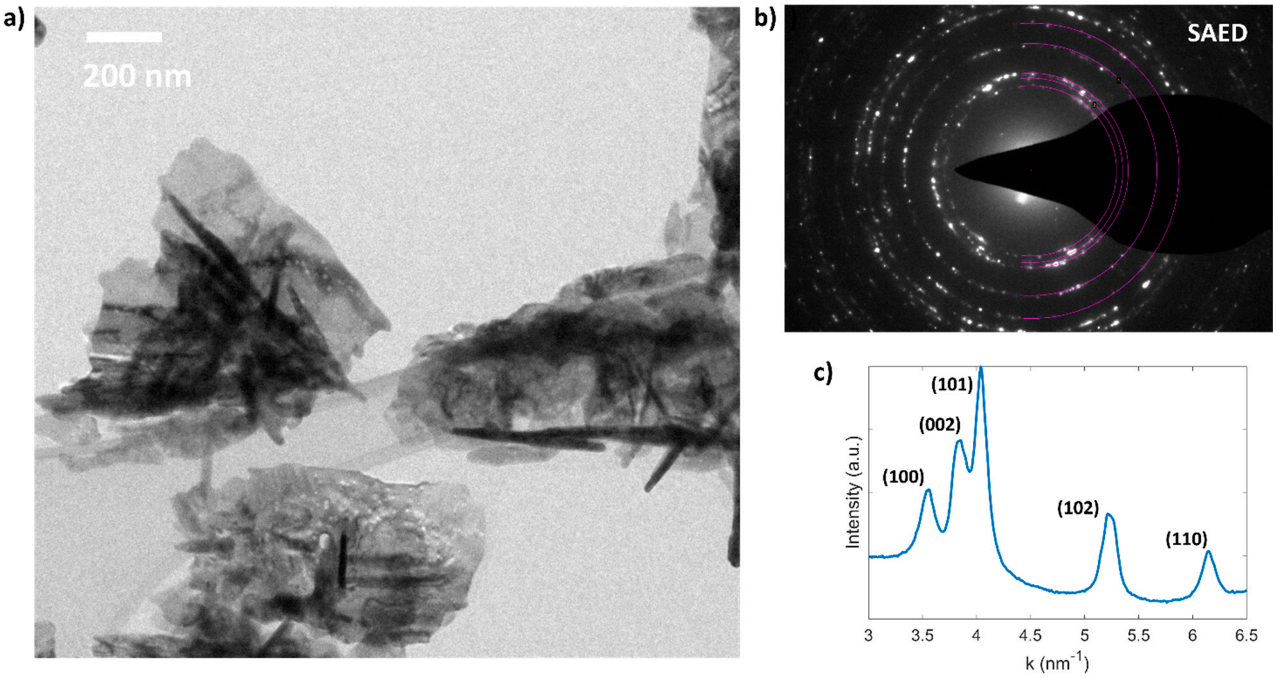

Figure S2. TEM characterizaion of sample ZNP 4: bright-field TEM image (a), selected area electron diffraction (SAED) pattern (b), rotationally-averaged electron diffraction pattern (c), with peak labels corresponding to family of planes characteristic of the ZnO wurtzite crystalline structure.

Structural information obtained by electron diffraction (Figure S2b, c) confirms the presence of ZnO wurtzite crystalline structure (hexagonal, *P63mc* space group) without secondary crystalline phases. The analysis of experimentally-obtained electron diffraction patterns (Figure S2b) was conducted with the Circular Hough analysis tool [D.R.G. Mitchell, Circular Hough transform diffraction analysis: A software tool for automated measurement of selected area electron diffraction patterns within Digital Micrograph™, Ultramicroscopy, 2008, 108(4), 367-374. Doi:10.1016/j.ultramic.2007.06.003] provided in Digital Micrograph for the identification of the diffraction rings. Subsequently, the rotationally-averaged electron diffraction pattern (Figure S2c) is obtained, where all the peaks can be ascribed to the ZnO crystalline phase.

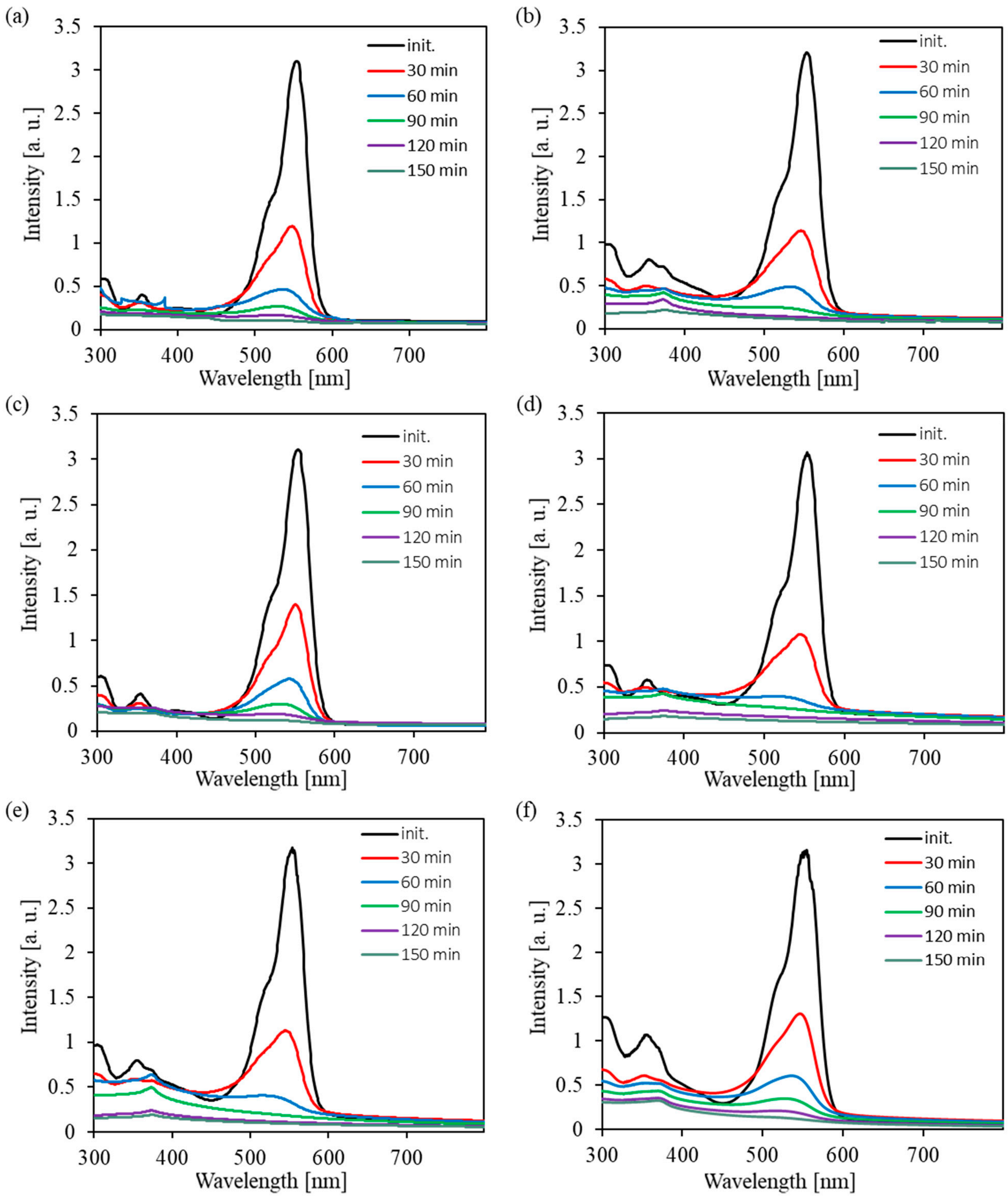

Figure S3. The optical density spectra of RhB solution under UV-Vis light illumination at different exposure time. The solution contained also ZnO nanostructures corresponding to samples (a) ZNP 1, (b) ZNP 2, (c) ZNP 3, (d) ZNP 4, (e) ZNP 5 and (f) ZNP 6.

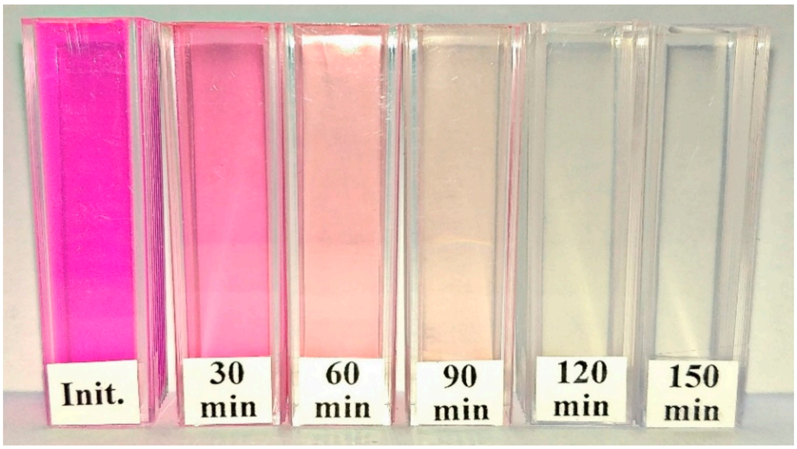

Figure S4. The change of color of the RhB solution with ZNP 4 for 150 min under UV-Vis illumination.

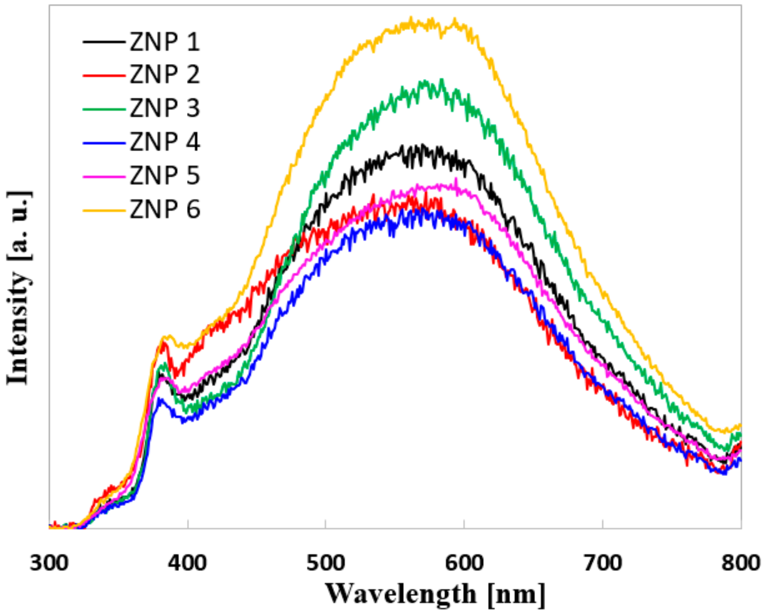

Figure S5. PL spectra of the synthesized ZnO samples taken at room temperature with excitation at a wavelength of 300 nm.

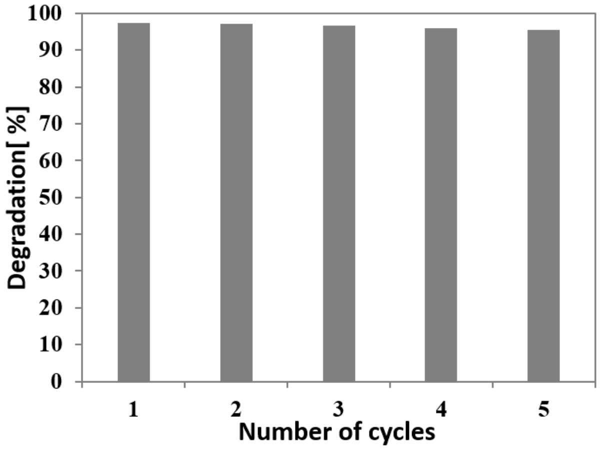

Figure S6. Reuse activity of ZNP 4 for RhB dye photodegradation.

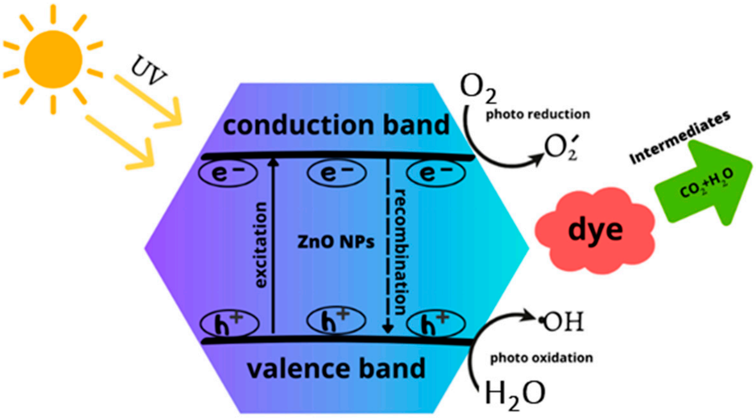

Figure S7. The proposed photocatalytic mechanism of ZnO nanostructures.

Table S1. Element percentages obtained by EDX analysis.

Sample name	Zinc (at.%)	Oxygen (at.%)	Carbon (at.%)
**ZNP 1**	48.9	45.2	5.9
**ZNP 2**	46.6	47.0	6.4
**ZNP 3**	48.3	45.7	6.0
**ZNP 4**	47.4	46.7	5.9
**ZNP 5**	45.1	48.6	6.3
**ZNP 6**	41.9	45.3	12.8

Table S2. Efficiency of photocatalytic degradation of dye in the presence of ZnO samples.

**Sample name**	R^* **after 150 min (%)**	K **(h^{-1})**
**ZNP 1**	96.96	1.42
**ZNP 2**	97.08	1.53
**ZNP 3**	95.99	1.31
**ZNP 4**	97.36	1.73
**ZNP 5**	97.08	1.53
**ZNP 6**	93.61	1.23

Table S3. Comparison the performance of the herein studied ZnO with the reported ZnO photocatalysts in literature.

	Article	k_{av}, h^{-1}
1.	ZNP 4 **(this article)**	1.73
2.	ZNP 2 **(this article)**	1.53
3.	ZNP 6 **(this article)**	1.23
4.	Guaraldo, T.T.; Wenk, J.; Mattia, D. Photocatalytic ZnO Foams for Micropollutant Degradation. Adv. Sustainable Syst. **2021**, *5*, 2000208. https://doi.org/10.1002/adsu.202000208	0.0182
5.	Aljaafari, A. Size Dependent Photocatalytic Activity of ZnO Nanosheets for Degradation of Methyl Red. Frontiers in Materials **2020**, *7*, 1-7. https://doi.org/10.3389/fmats.2020.562693	1.411
6.	Dasa, A.; Nai, R. G. Effect of aspect ratio on photocatalytic performance of hexagonal ZnO nanorods. Journal of Alloys and Compounds **2019,** 1-29. https://doi.org/10.1016/j.jallcom.2019.153277	1.542
7.	Tian, Ch.; Zhang, Q.; Wu, A.; Jiang, M.; Liang, Zh.; Jiang, B.; Fu, H. Cost-effective large-scale synthesis of ZnO photocatalyst with excellent performance for dye photodegradation. Chem. Commun. **2012**, *48*, 2858–2860. https://doi.org/10.1039/C2CC16434E	1.291
8.	Umar, K.; Mfarrej, M. F. B.; Rahman, Q. I.; Zuhaib, M.; Khan, A.; Zia, Q.; Banawas, S.; Nadeem, H.; Khan M. F.; Ahmad, F. ZnO Nano-swirlings for Azo Dye AR183 photocatalytic degradation and antimycotic activity. Scientific Reports **2022**, *12*, 14023. https://doi.org/10.1038/s41598-022-17924-3	0.906
9.	Zeljkovic, S.; Balaban, M.; Gajic, D.; Jelić D. Mechanochemically induced synthesis of N-ion doped ZnO: solar photocatalytic degradation of methylene blue. Green Chemistry Letters and Reviews **2022**, 1-13. https://doi.org/10.1080/17518253.2022.2108343	1.171
10.	Blažeka, D.; Radiˇci, R.; Maleti´c, D.; Živkovi, S. Enhancement of Methylene Blue Photodegradation Rate Using ZnO Nanoparticles. Nanomaterials **2022**, *12*, 2677. https://doi.org/10.3390/nano12152677	1.381
11.	Etay, H.; Kumar, A.; Yadav, O. P. Kinetics of photocatalytic degradation of methylene blue dye in aqueous medium using ZnO nanoparticles under UV radiation. J. Anal. Pharm. Res. **2023,** *12*, 32–37. https://doi.org/10.15406/japlr.2023.12.00421	0.3738
12.	Yusha'u, A.; Darma, M.S.; Isah, K.A. Sol-gel synthesis of ZnO nanoparticles for optmized photocatalytic degradation of Eriochrome Black T under UV irradiation. Alger. J. Eng. Technol. **2023**, *8*, 117-30. https://doi.org/10.57056/ajet.v8i1.100	1.291
13.	Akram, R.; Fatima, A.; Almohaimeed, Z. M.; Farooq, Z.; Qadir, K. W.; Zafar, Q.; Hua, M. Photocatalytic Degradation of Methyl Green Dye Mediated by Pure and Mn-Doped Zinc Oxide Nanoparticles under Solar Light Irradiation. Adsorption Science & Technology **2023**, *2023*, 1-15. https://doi.org/10.1155/2023/5069872	1.07

Table S4. Comparison of Zn-based electrocatalysts for CO_2 electrolysis in a batch cell.

**Materials**	**Potential (V *vs* RHE)**	**Electrolyte**	**Current density$_{CO}$ (mA·cm^{-2})**	**FE$_{CO}$ (%)**
ZnO [(1)]	-0.8	0.1 M $KHCO_3$	~ 0.9	~ 55
ZnO [(1)]	-0. 9	0.1 M $KHCO_3$	~ 2.0	~ 68
ED Zn dendrite[a (2)]	-0.9	0.5 M $NaHCO_3$	~ 4.0	~ 62
ED Zn[a (3)]	-1.0	0.1 M $KHCO_3$	~ 0.3	~ 62
Nanowire-like Zn [(4)]	-0.9	0.5 M $KHCO_3$	~ 30	~ 81
ED Zn[a (5)]	-0.9	0.5 M $KHCO_3$	~ 7.3	~ 73

ED ZnO[a] (6)	-0.9	0.5 M $KHCO_3$	~ 5.3	~77
ED Zn-3[a] (7)	-0.9	0.1 M $KHCO_3$	~ 4.9	~ 43
Zn NSs (8)	-0.9	0.1 M $KHCO_3$	~ 4.9	~ 78
ED P-Zn[a] (9)	-0.9	0.1 M $KHCO_3$	~ 23	~ 93
Zn/rGO (10)	-0.9	0.5 M $KHCO_3$	~ 2.1	~ 85
		**This study**		
ZNP 4	-1.2	0.1 M $KHCO_3$	~4.9	~ 84.3
ZNP 4	-1.0	0.2 M $KHCO_3$	~2.3	~ 70.2

[a] ED refers to electrodeposited route.

(1) Zeng, J.; Rino, T.; Bejtka, K.; Castellino, M.; Sacco, A.; Farkhondehfal, M. A.; Chiodoni, A.; Drago, F.; Pirri, C. F. Coupled copper-zinc catalysts for electrochemical reduction of carbon dioxide. *ChemSusChem* **2020**, *13*, 4128–4139, DOI 10.1002/cssc.202000971.

(2) Rosen, J.; Hutchings, G. S.; Lu, Q.; Forest, R. V.; Moore, A.; Jiao, F. Electrodeposited Zn dendrites with enhanced CO selectivity for electrocatalytic CO_2 reduction. *ACS Catal.* **2015**, *5* (8), 4586–4591, DOI 10.1021/acscatal.5b00922.

(3) Ren, D.; Ang, B. S.; Yeo, B. S. Tuning the selectivity of carbon dioxide electroreduction toward ethanol on oxide-derived Cu x Zn catalysts. *ACS Catal.* **2016**, *6* (12), 8239–8247, DOI 10.1021/acscatal.6b02162.

(4) Li, Y. H.; Liu, P. F.; Li, C.; Gui, H. Sharp-tipped zinc nanowires as an efficient electrocatalyst for carbon dioxide reduction. *Chem. - A Eur. J.* **2018**, 15486–15490, DOI 10.1002/chem.201803015.

(5) Lu, Y.; Han, B.; Tian, C.; Wu, J.; Geng, D.; Wang, D. Efficient electrocatalytic reduction of CO2 to CO on an electrodeposited Zn porous network. *Electrochem. commun.* **2018**, *97*, 87–90, DOI 10.1016/j.elecom.2018.11.002.

(6) Nguyen, D. L. T.; Jee, M. S.; Won, D. H.; Jung, H.; Oh, H. S.; Min, B. K.; Hwang, Y. J. Selective CO_2 reduction on zinc electrocatalyst: the effect of zinc oxidation state Induced by pretreatment environment. *ACS Sustain. Chem. Eng.* **2017**, *5* (12), 11377–11386, DOI 10.1021/acssuschemeng.7b02460.

(7) Qin, B.; Li, Y.; Fu, H.; Wang, H.; Chen, S.; Liu, Z.; Peng, F. Electrochemical reduction of CO2 into tunable syngas production by regulating the crystal facets of earth-abundant Zn catalyst. *ACS Appl. Mater. Interfaces* **2018**, *10* (24), 20530–20539, DOI 10.1021/acsami.8b04809.

(8) Li, C.; Shen, G.; Zhang, R.; Wu, D.; Zou, C.; Ling, T.; Liu, H.; Dong, C.; Du, X. Zn nanosheets coated with a ZnS subnanometer layer for effective and durable CO_2 reduction. *J. Mater. Chem. A* **2019**, *7* (4), 1418–1423, DOI 10.1039/C8TA10799H.

(9) Luo, W.; Zhang, J.; Li, M.; Züttel, A. Boosting CO production in electrocatalytic CO_2 reduction on highly porous Zn catalysts. *ACS Catal.* **2019**, *9* (5), 3783–3791, DOI 10.1021/acscatal.8b05109.

(10) Nguyen, D. L.; Lee, C. W.; Na, J.; Kim, M.; Tu, N. D. K.; Lee, S. Y.; Sa, Y. J.; Won, D. H.; Oh, H.; Kim, H.; Min, B. K.; Han, S. S.; Lee, U.; Hwang, Y. J. Mass transport control by surface graphene oxide for selective CO production from electrochemical CO_2 reduction. *ACS Catal.* **2020**, *10*, 3222–3231, DOI 10.1021/acscatal.9b05096.
